# Infertility Distress Management in Couples Treated with Assisted Reproductive Techniques (ART) in COVID-19 Pandemic

**DOI:** 10.18502/jri.v21i4.4337

**Published:** 2020

**Authors:** Fatemeh Hamidi, Farzaneh Babapour, Zeinab Hamzehgardeshi

**Affiliations:** 1- M.Sc., Student of Midwifery Counseling, Student Research Committee, Department of Midwifery, Nasibeh School of Nursing and Midwifery, Mazandaran University of Medical Sciences, Sari, Iran; 2- Sexual and Reproductive Health Research Center, Department of Reproductive Health and Midwifery, School of Nursing and Midwifery, Mazandaran University of Medical Sciences, Sari, Iran

The respiratory disease caused by a new coronavirus was first observed in Wuhan, Hubei Province, China in December 2019. The coronavirus disease (Covid-19) spread rapidly with human-to-human transmission around the world and raised global concerns, and it caused the World Health Organization (WHO) to consider it as a pandemic. Currently, the lives of many people around the world are affected by the crisis caused by the outbreak of Covid-19 disease, which despite efforts in many countries to reduce the risk and negative effects of this crisis, the damage and the resulting costs are increasing (1). This crisis may affect infertile couples, and it may have a negative impact on the mental health and all aspects of an individual’s life (2). Infertility is a disease of the genital tract that is characterized by a lack of pregnancy after twelve months or more of having a regular unprotected sex (3). The World Health Organization estimates that one in six couples will experience a delay in pregnancy, and that an increasing number will require treatment with assisted reproductive techniques. Statistically about 9% of couples all around the world will experience infertility during their lifetime (4). Infertility is one of the most stressful and sensitive experiences that threatens the stability of the individual, family, marriage and society, which will be more threatening in crisis situations (5). Assisted reproductive techniques (ARTs) are a common treatment of choice for many infertile couples due to male or female or idiopathic factors (6). There are various methods of mental disorders management in pregnant women such as CBT, acupuncture, and food supplements (7). For overcoming the stress during the outbreak of such diseases such as influenza and SARS, there are ways to manage such anxiety such as psychological intervention by using therapeutic dialogues, inducing hope, listening and support, social support and face-to-face counseling which should be offered by the healthcare workers such as the nursing and medical staff (8). Due to the lack of information about the new coronavirus and its behaviors, various methods are used by infertility treatment centers. Some health centers recommend that patients who are undergoing a new IVF cycle or recently have started taking their relevant medications should continue their treatments. On the other hand, some associations in American, British and European societies all emphasize that due to the unknown effects of coronavirus on the fetus and pregnancy and considering emergency conditions for public health service providers, no fertility treatment cycle should be started at present. This means that no ovulation, IUI, fresh IVF or frozen embryo transfer cycles should be performed. Due to the decrease in number of visits and counseling with infertile couples in clinics, the mental health issues and the strategies for reducing distresses of infertile couples are of paramount importance. Since infertile couples are also more vulnerable to disease, it is important to pay attention to their health. Delays in fertility treatment during the Covid-19 pandemic can cause stress and anxiety and it’s completely understandable. Stress and anxiety can alter the balance of hormones needed for pregnancy. It can also affect the success of a couple's pregnancy in other ways (9–11). A review of literatures shows that currently couples are getting help by using a variety of methods, such as online counseling through social networks like Skype to receive virtual counseling, support, and assistance. Couples can also sign a consent form and, if necessary, receive their treatment protocol online at the patient's portal. If Covid-19 worries people about their treatment, talking to a fertility counselor, fertility coach and health care provider can help couples feel more in control of their life during this confusing period. Telemedicine and psychological support have established a good tool for ART specialists. Most fertility organizations recommended the use of such tools during the pandemic (9, 12). Lack of preparedness, stress, anxiety and failing to cope with this kind of crisis cause a lot of psychological damage to infertile couples, which is irreparable. Therefore, for distress management, health care providers can use a variety of safe methods such as online counseling, online supports, social networks, peer supports, thereby continuing treatment with a full explanation of the existing conditions and giving the right of choice to couples, especially for infertile couples who are being treated with assisted reproductive techniques (ARTs) in Covid-19 pandemic.
